# Family history of hematologic malignancies and risk of multiple myeloma: differences by race and clinical features

**DOI:** 10.1007/s10552-015-0685-2

**Published:** 2015-11-23

**Authors:** MaryAnn E. VanValkenburg, Gwendolyn I. Pruitt, Ilene K. Brill, Luciano Costa, Maryam Ehtsham, Ian T. Justement, Racquel D. Innis-Shelton, Donna Salzman, E. Shyam P. Reddy, Kelly N. Godby, Fady M. Mikhail, Andrew J. Carroll, Vishnu B. Reddy, Ralph D. Sanderson, Louis B. Justement, Paul W. Sanders, Elizabeth E. Brown

**Affiliations:** Department of Pathology, School of Medicine, University of Alabama at Birmingham, Birmingham, AL 35294-3300 USA; Department of Epidemiology, School of Public Health, University of Alabama at Birmingham, Birmingham, AL USA; Division of Hematology and Medical Oncology, Department of Medicine, School of Medicine, University of Alabama at Birmingham, Birmingham, AL USA; UAB Comprehensive Cancer Center, University of Alabama at Birmingham, Birmingham, AL USA; School of Medicine, University of Alabama at Birmingham, Birmingham, AL USA; Cancer Biology Program, Department of Obstetrics and Gynecology, Morehouse School of Medicine, Atlanta, GA USA; Department of Genetics, School of Medicine, University of Alabama at Birmingham, Birmingham, AL USA; Department of Microbiology, School of Medicine, University of Alabama at Birmingham, Birmingham, AL USA; Division of Nephrology, School of Medicine, University of Alabama at Birmingham, Birmingham, AL USA; Department of Veterans Affairs Medical Center, Birmingham, AL USA; 1824 6th Avenue South, WTI 602C, Birmingham, AL 35294-3300 USA

**Keywords:** Multiple myeloma, Family history, Genetic, Black, African American

## Abstract

**Purpose:**

Multiple myeloma (MM) is the most common hematologic malignancy affecting Blacks in the USA, with standardized incidence rates that are twofold to threefold higher than Whites. The rationale for the disparity is unclear.

**Methods:**

Using participants enrolled in the Molecular And Genetic Epidemiology study of myeloma (259 MM cases; 461 controls), we examined the risk of MM associated with family history of cancer, differences by race and among cases, defining clinical features. Risk estimates were calculated using odds ratios and corresponding 95% confidence intervals from logistic regression adjusted for confounders.

**Results:**

Overall, MM risk in cases with relatives affected with any hematologic malignancy was significantly elevated compared to controls (OR 1.89, 95% CI 1.25–2.86). Myeloma risk associated with a family history of MM was higher than the risk associated with any hematologic malignancy (OR 3.75, 95% CI 1.75–8.05), and the effect was greater for Blacks (OR 20.9, 95% CI 2.59–168) than Whites (OR 2.04, 95% 0.83–5.04), among cases with early onset (≤60 years; OR 4.58, 95% CI 1.21–17.3) and with increasing numbers of affected relatives (*p* trend = 0.001). Overall, frequencies of end organ damage differed in cases with relatives affected with any hematologic malignancy and significantly more cases exhibited κ light chain restriction (OR 3.23, 95% CI 1.13–9.26).

**Conclusions:**

The excess risk of MM observed in Blacks and the variation in clinical features observed in MM patients according to family history of hematologic malignancy may be attributed to a shared germline and environmental susceptibility.

**Electronic supplementary material:**

The online version of this article (doi:10.1007/s10552-015-0685-2) contains supplementary material, which is available to authorized users.

## Introduction

Multiple myeloma is a plasma cell malignancy characterized, in part, by prolonged survival and accumulation of clonal plasma cells in the bone marrow microenvironment, presence of monoclonal protein in serum, urine or both, and end organ damage [1]. Standardized incidence rates of MM are increasing, advancing it to the second most common hematologic malignancy and accounting for 1 % of all cancers in the USA [2]. Although the etiology of MM is unclear, it is preceded by an asymptomatic plasma cell dyscrasia known as Monoclonal Gammopathy of Undetermined Significance (MGUS) [3, 4] that carries a risk of progression to frank MM of 1 % per year [5]. Other confirmed risk factors for MM include increasing age, male sex, Black race and a family history of cancer [6].

Multiple myeloma is the most common hematologic malignancy affecting Blacks in the USA, with standardized incidence rates that are twofold to threefold higher than Whites [7, 8], and with an earlier age of onset [9]. Rationale for the observed disparity is unclear. However, evidence suggests a shared genetic predisposition.

Several lines of evidence support an inherited germline susceptibility. Familial clustering of MM in several case series [10–13], in addition to family aggregation [14, 15], epidemiologic case–control [16, 17], and registry-based [18, 19] studies have consistently shown excess MM risk among first-degree relatives of patients with MM. In addition, in the only study published to date that included both Blacks and Whites, Brown et al. [20] showed that MM risk was significantly increased in Black MM patients with an affected first-degree relative, providing a possible rationale for the difference in incidence observed by race.

Familial aggregation of MM and the epidemiologic differences observed by race suggest a complex etiology, which may be influenced by shared genetic factors, environmental exposures, behaviors and underlying differences in tumor biology. We conducted a comprehensive investigation to expand upon the existing report to evaluate differences in the contribution of hematologic malignancies and solid tumors among relatives of Black and White patients with MM. To our knowledge this is the first study to include evaluations of MM-defining clinical features with family history of cancer, which may provide important insight into underlying differences in the clinical presentation of MM by race.

## Materials and methods

### Study population

We included participants enrolled in the Molecular And Genetic Epidemiology (iMAGE) study of myeloma to characterize the contribution of family history of cancer on the risk of MM, differences by race and among cases only, the presence of defining clinical features. The iMAGE study was designed to evaluate the effects of biological, chemical, physical, social and genetic influences on the risk of MM and direct comparisons by self-reported Black and White race. Approvals from the appropriate Institutional Review Boards in accordance with the Declaration of Helsinki were obtained prior to study initiation, and informed consent was obtained from all individual participants included in the study.

#### Case definition

Eligible cases were recruited from the University of Alabama at Birmingham Hematology and Medical Oncology clinics (Birmingham, Alabama) and the Morehouse School of Medicine (Atlanta, Georgia). Patients with a diagnosis of MM were identified based on the ICD-9 classifications (203) or International Classification of Diseases for Oncology third revision code 9732/3 and confirmed based on the revised and updated International Multiple Myeloma Working Group classification criteria for MM. Criteria include the cumulative presence of clonal bone marrow plasma cells ≥10 % or biopsy proven bony or extramedullary plasmacytoma and presence of one or more MM-defining events including organ damage (hypercalcemia, renal insufficiency, anemia, or lytic bone lesions or severe osteopenia or pathologic fractures attributed to a plasma cell proliferative disorder), or in the absence of end organ damage, clonal bone marrow plasma cells ≥60 %, serum free light chain (FLC) ratio ≥100, or more than one focal bone lesion (>5 mm) identified using magnetic resonance imaging (MRI) [21]. Each MM case was reviewed by an expert panel to ensure consistent case definitions and to minimize phenotype misclassification.

#### Clinical features

Diagnostic and defining clinical features including clonal bone marrow plasma cells (%), serum monoclonal (M)-protein (median, range), abnormal FLC ratio (<0.26 or >1.65), immunoglobulin (Ig) isotype (IgA, IgG, IgM, IgD, biclonal), clonality (kappa, lambda, biclonal), β_2_-microglobulin (median, range), albumin (median, range), end organ damage [presence of hypercalcemia (serum calcium, >11.5 mg/dL), renal insufficiency (serum creatinine, >177.0 μmol/L (>2 mg/dL) or estimated creatinine clearance <40 mL/min per 1.73 m^2^), anemia (normochromic, normocytic with hemoglobin >2 g/dL below the lower limit of normal or hemoglobin <10 g/dL), bone lesions (radiologic evidence of lytic lesions, severe osteopenia or pathologic fractures)] [21], and the revised and updated International Staging System (ISS) [22] were determined by laboratory studies, medical history, and physical examination, respectively.

#### Control selection

Controls were sampled from an existing and updated population-based database established and maintained by the Survey Research Unit (University of Alabama at Birmingham). This database includes US Census and Centers for Disease Control population databases established from list-assisted random digit dialing methods and used previously for this, and other large-scale population-based epidemiology studies [23, 24]. Eligible controls were residents of Alabama and Georgia, 21 years of age or older without a self-reported history of MGUS, smoldering myeloma (SMM), MM, or other cancer excluding non-melanoma cancers of the skin. One to two controls were randomly selected and frequency matched to cases on age (±5 years), sex, race (Black, White), and geography.

### Definition of family history of cancer

Detailed information, including family history of cancer, sociodemographic features, smoking and alcohol use, medication use, as well as residential, lifetime occupational, medical, surgical, and reproductive histories, was obtained using a structured questionnaire administered by trained interviewers at the time of enrollment. We defined family history of cancer as a self-report of one or more first-degree (parent, sibling, child), second-degree (grandparent, aunt, uncle, niece, nephew), or third-degree (first-cousin) relatives with any hematologic malignancy including MM, non-Hodgkin lymphoma [NHL; which included lymphoma not otherwise specified (NOS)], Hodgkin lymphoma (HL), leukemia, or any solid tumor (non-hematologic malignancy). Family history of any hematologic malignancy was defined using ICD-9 classification including MM (203), NHL (202), HL (201), or leukemia (204–208). As a sensitivity assessment, MM was defined with and without self-reported affected relatives with bone cancer NOS and later excluded from the MM definition to minimize misclassification. We categorized affected relatives as first-degree and jointly as any relative. Family size was not collected.

### Statistical analysis

We evaluated family history of cancer with MM risk overall and stratified by race, early age of onset (≤60 years, defined by median) and sex of the MM case as well as the affected relative to evaluate sex-linked germline susceptibility. Among cases only, we evaluated family history of cancer with the presence of defining clinical features. We estimated the risk of MM (case–control analysis) and risk of family history of cancer in MM patients (case-only analysis) using the odds ratio (OR) and corresponding 95% confidence interval (CI) calculated from logistic regression adjusted for confounders including sex, age (continuous), level of education (≤high school graduate vs. some college, college graduate, or post-graduate education) and race (White, Black) in analyses not stratified by these variables. Other potential confounders were evaluated, including smoking status, alcohol consumption, and annual household income at the time of enrollment, but were excluded from final models because they were not substantially related to MM or family history of cancer. Tests for statistical significance of trend were conducted using multivariable logistic regression with an incremental increase in the number of affected relatives per category modeled as a continuous variable. The strength of linearity between clinical laboratory variables and a family history of any hematologic malignancy among MM cases was examined using regression coefficients and standard errors generated by linear regression adjusted for confounders. Statistical significance, based on multivariable logistic models, was calculated using the maximum likelihood *χ*^2^ test, and differences between strata were determined using the Mantel–Haenszel *χ*^2^ test for homogeneity. Individuals with missing data for family history of cancer variables or clinical features were excluded from analyses. A two-sided *p* value ≤0.05 was considered statistically significant. All analyses were conducted using SAS version 9.4 (Cary, NC).

## Results

From May 2009 to May 2013, the iMAGE study team constituted the population-based, frequency-matched, case–control study that includes a total of 790 participants (277 cases and 513 controls). Of the 344 eligible cases, 167 (83.9 %) Whites and 110 (75.9 %) Blacks were enrolled (overall case participation rate, 80.5 %). Reasons for refusal to participate include, refused to be interviewed (2.5 % Whites, 8.3 % Blacks), patient too ill (2.0 % Whites, 0.7 % Blacks), or other (11.6 % Whites, 15.2 % Blacks). Cases with extramedullary or solitary plasmacytoma (without evidence of end organ damage, clonal bone marrow plasma cells ≥60 %, FLC ratio >100 or focal bone lesion >5 mm by MRI), amyloidosis, Waldenström Macroglobulinemia, monoclonal immunoglobulin deposition disease, Polyneuropathy Organomegaly Endocrinopathy Edema M-protein Syndrome, (POEMS), MGUS and HIV-1 seropositivity were excluded (*n* = 17). An additional case withdrew participation and was terminated from the study. After initial eligibility screening, participation rates for controls were 80.8 % (79.7 % for Whites and 82.3 % for Blacks). Enrolled controls later discovered to have MGUS (*n* = 1), be duplicates (*n* = 2), related to a case (*n* = 4), reported a shared residential area with a case or other enrolled control for 2 or more years (*n* = 32) and with reported diagnoses of cancer, myelodysplastic syndrome (*n* = 7), HIV-1 infection (*n* = 4) or solid organ transplant (*n* = 2) were excluded leaving a total of 259 cases and 461 controls available for analysis.

Distributions of demographic characteristics of participants enrolled in the iMAGE study of myeloma are shown in Table 1. In the combined population, cases and controls did not differ substantially by race; however, modest non-clinically significant differences were observed by age and sex despite frequency matching on these factors, of which, the latter is indicative of a disproportionately higher participation rate among female controls. Of the total 259 cases, the majority were male (54.8 %) with a mean age of 60 years at the time of diagnosis. Black cases were significantly younger at diagnosis compared to White cases (mean age, 58 vs. 61 years; *p* = 0.005) and Black cases reported less education (*p* = 0.006), annual household income (*p* = 0.004) and fewer relatives affected with any cancer (*p* = 0.0002) than their White counterparts.

**Table 1:** Characteristics of participants enrolled in the Molecular And Genetic Epidemiology (iMAGE) study of myeloma, overall and stratified by race

Demographic characteristics	White		Black	
	Case	Control	Case	Control
No. of persons (%)	154 (59.4)	263 (57.1)	105 (40.5)	198 (43.0)
Mean age, years (SD)^a^	61.3 (8.2)	64.9 (10.2)	58.0 (10.4)	59.2 (11.9)
Sex, *n* (%)^a^				
Male	99 (64.3)	142 (54.0)	43 (41.0)	67 (33.8)
Female	55 (35.7)	121 (46.0)	62 (59.1)	131 (66.2)
Education, *n* (%)				
High school graduate or less	34 (22.4)	41 (15.6)	40 (38.1)	49 (24.7)
Some college, college graduate or post-graduate education	118 (77.6)	222 (84.4)	65 (61.9)	149 (75.3)
Smoking status, *n* (%)^b^				
Never smoker	85 (55.2)	121 (46.0)	57 (54.3)	98 (50.0)
Ever smoker	69 (44.8)	142 (54.0)	48 (45.7)	98 (50.0)
Alcohol consumption, *n* (%)^c^				
Never drinker	79 (51.3)	126 (47.9)	62 (59.0)	112 (56.6)
Ever drinker	75 (48.7)	137 (52.1)	43 (41.0)	86 (43.4)
Annual household income at enrollment, *n* (%)				
Less than 20,000	14 (14.1)	24 (11.5)	20 (28.2)	50 (35.7)
20,000–29,999	10 (10.1)	26 (12.4)	13 (18.3)	29 (20.7)
30,000–49,999	23 (23.2)	40 (19.1)	19 (26.8)	36 (25.7)
50,000–99,999	27 (27.3)	70 (33.5)	13 (18.3)	19 (13.6)
100,000 or more	25 (25.3)	49 (23.4)	6 (8.5)	6 (4.3)
No. relatives with cancer, mean (SD)	2.8 (2.4)	2.0 (1.7)	1.8 (1.5)	1.5 (1.7)

^a^Among European Americans, controls were significantly older than cases (65 vs. 61 years, respectively; *p* = 0.002), albeit this difference is not clinically significant and falls within the expected range of values based on frequency matching ±5 years. In addition, in the total population, the proportion of male controls to cases was modestly lower than in females (*p* = 0.04). No other comparison in the total population reached a level of statistical significance, *p* > 0.05

^b^Smoking status (ever smoker) defined as having smoked more than 100 cigarettes in a lifetime

^c^Alcohol consumption (ever drinker) defined as at least one alcoholic beverage (beer, wine, hard liquor) per week for 6 months or longer

The estimated risk of MM associated with a family history of cancer is shown in Table 2. In the combined population, the majority of participants reported a family history of cancer (79.9 %), including any solid tumor (74.3 %) and any of the combined four hematologic malignancies (NHL, HL, leukemia and MM; 16.4 %). Among controls with any relative affected with any hematologic malignancy, family history of leukemia was the most prevalent (*n* = 32; 7 %) followed by NHL (*n* = 20; 4 %), MM (*n* = 11; 2 %) and HL (*n* = 6; 1 %), consistent with the prevalence of these hematologic malignancies in the general US population.

**Table 2:** Frequencies and risk estimates of multiple myeloma associated with family history of cancer, overall and stratified by race

	White				Black				Total population	
	Case, *n* (%)	Control, *n* (%)	OR (95% CI)^b^	*p*	Case, *n* (%)	Control, *n* (%)	OR (95% CI)^b^	*p*	OR (95% CI)^c^	*p*
No family history of cancer^a^	17 (11.1)	42 (16.1)	1.0 (reference)		20 (19.2)	61 (31.0)	1.0 (reference)		1.0 (reference)	
Any cancer	136 (88.9)	219 (83.9)	1.84 (0.97–3.48)	0.062	84 (80.8)	136 (69.0)	2.15 (1.18–3.90)	0.012	1.97 (1.28–3.04)	0.002
Any hematologic malignancy	41 (26.8)	46 (17.6)	1.77 (1.08–2.91)	0.022	16 (15.4)	15 (7.6)	2.43 (1.13–5.22)	0.023	1.89 (1.25–2.86)	0.002
Multiple Myeloma	11 (7.2)	10 (3.8)	2.04 (0.83–5.04)	0.120	9 (8.7)	1 (0.5)	20.9 (2.59–168)	0.004	3.75 (1.75–8.05)	0.001
Hematologic malignancy excluding myeloma	34 (22.2)	38 (14.6)	1.71 (1.01–2.89)	0.047	8 (7.7)	15 (7.6)	1.09 (0.44–2.71)	0.855	1.48 (0.94–2.32)	0.090
Non-Hodgkin lymphoma (NHL)	13 (8.5)	15 (5.8)	1.71 (0.78–3.76)	0.179	2 (1.9)	5 (2.5)	0.81 (0.15–4.33)	0.801	1.47 (0.73–2.97)	0.283
Hodgkin lymphoma (HL)	3 (2.0)	3 (1.2)	1.81 (0.36–9.20)	0.474	2 (1.9)	3 (1.5)	1.53 (0.25–9.42)	0.647	1.65 (0.49–5.50)	0.418
Leukemia	20 (13.1)	23 (8.8)	1.52 (0.78–2.94)	0.218	4 (3.9)	9 (4.6)	0.84 (0.25–2.85)	0.777	1.26 (0.71–2.23)	0.427
Any solid tumor	127 (83.0)	207 (79.3)	1.56 (0.90–2.71)	0.112	74 (71.2)	127 (64.5)	1.55 (0.91–2.66)	0.107	1.55 (1.06–2.27)	0.024
Prostate	28 (18.3)	42 (16.1)	1.27 (0.74–2.19)	0.384	21 (20.2)	37 (18.8)	1.16 (0.63–2.13)	0.628	1.23 (0.82–1.83)	0.323
Lung	41 (26.8)	61 (23.4)	1.16 (0.72–1.85)	0.545	26 (25.0)	41 (20.8)	1.37 (0.77–2.42)	0.282	1.24 (0.87–1.78)	0.239
Breast	51 (33.3)	80 (30.7)	1.21 (0.78–1.88)	0.396	32 (30.8)	62 (31.5)	1.07 (0.63–1.80)	0.814	1.15 (0.83–1.61)	0.403
Colon	28 (18.3)	43 (16.5)	1.41 (0.82–2.43)	0.215	9 (8.7)	30 (15.2)	0.54 (0.24–1.20)	0.132	1.01 (0.65–1.56)	0.984
Melanoma	10 (6.5)	14 (5.3)	1.22 (0.51–2.88)	0.657	0 (0.0)	1 (0.5)	–	–	1.21 (0.53–2.80)	0.652
Head and neck^d^	10 (6.5)	19 (7.3)	0.96 (0.43–2.16)	0.928	10 (9.6)	3 (1.5)	6.98 (1.85–26.4)	0.004	1.69 (0.90–3.19)	0.104
Gynecologic^e^	22 (14.4)	20 (7.7)	2.22 (1.15–4.30)	0.018	6 (5.8)	8 (4.1)	1.47 (0.49–4.46)	0.494	1.95 (1.11–3.43)	0.020
Genitourinary^f^	13 (8.5)	10 (3.8)	2.69 (1.12–6.46)	0.026	2 (1.9)	5 (2.5)	0.81 (0.15–4.31)	0.806	2.07 (0.98–4.37)	0.056

^a^Family history of cancer includes any first-degree (parent, sibling, child), second-degree (grandparent, aunt, uncle, niece, nephew) or third-degree (cousin) relative. Family history of cancer data was missing in a total of *n* = 5 participants (*n* = 2 cases and *n* = 3 controls)

^b^Adjusted for sex, age, and level of education (>high school graduate or less)

^c^Adjusted for sex, age, level of education (>high school graduate or less) and race (White, Black)

^d^Head and neck cancers include laryngeal, hypopharyngeal, nasal cavity, paranasal sinus, nasopharyngeal, oral, oropharyngeal, salivary glad and head and neck, NOS

^e^Gynecologic cancers include cervical, uterine, endometrial, ovarian, and gynecologic, NOS

^f^Genitourinary cancers include bladder, kidney, testicular, and urinary tract

In cases with any relative affected with any hematologic malignancy, the risk of MM was significantly elevated compared to controls (OR 1.89, 95% CI 1.25–2.86). The magnitude of this effect was greater in Blacks (OR 2.43, 95% CI 1.13–5.22) than in Whites (OR 1.77, 95% CI 1.08–2.91), although the difference in the magnitude of effect by race was not statistically significant (*p* = 0.532).

The risk of MM associated with a family history of MM was higher than the risk associated with any hematologic malignancy (OR 3.75, 95% CI 1.75–8.05), and this effect was greater for Blacks (OR 20.9, 95% CI 2.59–168) than Whites (OR 2.04, 95% 0.83–5.04). Although risk estimates are based in a small sample, these relationships were substantiated in an analysis restricted to participants who reported MM among first-degree relatives only (Blacks: OR 10.8, 95% CI 1.22–94.8; Whites: OR 1.19, 95% CI 0.28–5.16; data not shown). In contrast, increased risk of MM among cases with a family history of NHL, HL or leukemia (hematologic malignancy excluding MM) was present in Whites (OR 1.71, 95% CI 1.01–2.89), whereas no association was observed in Blacks.

Sample size precluded our ability to evaluate MM risk by race further stratified by sex or age. In the combined population, risks associated with a family history of MM were elevated among cases with two or more affected relatives with any cancer, any hematologic malignancy or MM (*p* trend ≥0.001) (Supplementary Table 1). In addition, the influence of a positive family history of myeloma had a greater magnitude of effect in patients with early age of onset (≤60 years of age; OR 4.58, 95% CI 1.21–17.3), although the difference by age strata was not statistically significant, and risk estimates were similarly elevated in males and females (Table 3).

**Table 3:** Risk estimates of multiple myeloma associated with family history of any cancer, any hematologic malignancy and multiple myeloma with early age of onset and sex

	Any cancer				Hematologic malignancy				Multiple myeloma			
	Case	Control	OR (95% CI)^a^	*p*	Case	Control	OR (95% CI)^a^	*p*	Case	Control	OR (95% CI)^a^	*p*
Age of onset												
<60 years	108 (85.7)	114 (70.4)	2.64 (1.42–4.93)	0.002	27 (21.4)	15 (9.3)	2.61 (1.30–5.23)	0.007	10 (7.9)	3 (1.9)	4.58 (1.21–17.3)	0.025
>60 years	112 (85.5)	241 (81.4)	1.57 (0.86–2.87)	0.141	30 (22.9)	46 (15.5)	1.67 (0.98–2.85)	0.061	10 (7.6)	8 (2.7)	3.38 (1.28–8.95)	0.014
Sex												
Male	120 (85.1)	154 (74.0)	2.11 (1.18–3.77)	0.012	31 (22.0)	28 (13.5)	1.77 (0.99–3.17)	0.053	10 (7.1)	5(2.4)	3.49 (1.15–10.5)	0.027
Female	100 (86.2)	201 (80.4)	1.87 (0.97–3.59)	0.062	26 (22.4)	33 (13.2)	2.00 (1.10–3.62)	0.023	10 (8.6)	6(2.4)	4.20 (1.45–12.2)	0.008

^a^Adjusted for sex, age, level of education (>high school graduate or less) and race (White, Black)

The estimated risk of MM associated with a family history of solid tumors is shown in Table 2. In the combined population, the risk of MM was modestly elevated with a family history of any solid tumor (OR 1.55, 95% CI 1.06–2.27) and for the combined category of gynecologic cancers (OR 1.95, 95% CI 1.11–3.43). Affected relatives with a history of head and neck cancer were strongly associated with MM risk only in Blacks (OR 6.98, 95% CI 1.85–26.4), whereas the excess risk among those with a family history of genitourinary cancers (excluding prostate) was present only in Whites (OR 2.69, 95% CI 1.12–6.46), albeit findings may be limited by sample size. Although the risk of MM was modestly elevated with a family history of a variety of solid tumors, no single solid tumor type included in any of the combined solid tumor categories achieved a level of statistical significance.

Differences in the distribution of clinical features of MM cases with and without a family history of any hematologic malignancy are shown in Table 4. Of the 57 MM cases with a family history of hematologic malignancy, kappa (κ) light chain restriction was detected in 43 (78.2 %) MM cases compared to 115 (64.3 %) MM cases without a family history of hematologic malignancy (*p* = 0.045). No notable difference in MM risk was observed for light chain MM (*p* = 0.616). However, in cases with heavy-chain MM, individuals with a family history of hematologic malignancies were more likely to exhibit IgG kappa MM, with a notable κ light chain restriction (OR 3.23, 95% CI 1.13–9.26; *p* = 0.029) after the heavy-chain isotype (IgG, IgA) was held constant. Of the diagnostic criteria for end organ damage, the presence of anemia and renal insufficiency, attributed to MM, was notably less frequent consistent with a twofold reduction in risk of MM in cases with a family history of hematologic malignancy compared to those without, whereas hypercalcemia and lytic bone lesions were more frequent, albeit not significantly (*p* ≥ 0.230). We found no other notable differences in the distributions of clinical characteristics among MM cases with and without a family history of hematologic malignancies. Insufficient sample size precluded our ability to evaluate MM-defining clinical features stratified by race.

**Table 4:** Clinical features and laboratory characteristics of multiple myeloma cases with and without a family history of any hematologic malignancy

Clinical feature and laboratory characteristic^a^	MM case with affected relative (*n* = 57)	MM case with unaffected relative (*n* = 200)	OR (95% CI)^b^	*p*
Clonal bone marrow plasma cells (BMPC), median (range) %	40 (3–97)	50 (1–100)	–	0.490
Serum monoclonal protein, median (range) g(dL)	2.6 (0.1–9.5)	2.6 (0.1–10.5)	–	0.442
Abnormal free light chain (FLC) ratio (<0.26 or >1.65), *n* (%)	28 (90.3)	101 (92.3)	0.68 (0.16–2.85)	0.597
Light chain disease, *n* (%)	12 (21.4)	42 (23.5)	0.83 (0.39–1.74)	0.616
Immunoglobulin (Ig)-G isotype, *n* (%)^c^	33 (75.0)	99 (74.4)	1.03 (0.46–2.78)	0.946
Kappa free light chain, *n* (%)^d^	43 (78.2)	115 (64.3)	2.08 (1.02–4.25)	0.045
β_2_-Microglobulin, median (range) mg/dL	3.3 (1.4–21.6)	3.9 (0.6–32.1)	–	0.701
Creatinine, median (range) mg/dL	1.1 (0.6–11.9)	1.2 (0.5–26.2)	–	0.236
Albumin, median (range) mg/dL	3.5 (1.6–4.9)	3.5 (1.6–4.8)	–	0.220
End organ damage, *n* (%)				
Hypercalcemia (serum calcium >11.5 mg/dL)	11 (23.4)	25 (16.0)	1.66 (0.73–3.77)	0.230
Renal insufficiency (serum creatinine >2 mg/dL)	7 (14.6)	41 (26.3)	0.48 (0.20–1.81)	0.111
Anemia (hemoglobin <10 or >2 g/dL below the lower limit of normal)	24 (48.0)	110 (69.6)	0.43 (0.22–0.83)	0.011
Bone lesions (lytic lesions, pathologic fractures or severe osteopenia)	43 (81.1)	127 (77.0)	1.32 (0.59–2.94)	0.495
Cumulative presence of end organ damage, *n* (%)				
1	26 (50.0)	72 (43.6)	1.0 (reference)	
2	21 (40.4)	62 (37.6)	1.01 (0.51–1.99)	0.977
3	3 (5.8)	17 (10.3)	0.52 (0.14–1.96)	0.336
4	2 (3.9)	14 (8.5)	0.39 (0.08–1.90)	0.244
ISS stage, *n* (%)				
Stage 1	12 (37.5)	43 (31.4)	1.0 (reference)	
Stage 2	11 (34.4)	51 (37.2)	0.75 (0.30–1.89)	0.543
Stage 3	9 (28.1)	43 (31.4)	0.78 (0.29–2.04)	0.606
Combined immunoglobulin isotype and light chain^e^				
IgG kappa	27 (64.3)	61 (46.6)	2.12 (1.02–4.42)	0.043
IgG lambda	5 (11.9)	36 (27.5)	0.35 (0.12–0.96)	0.042
IgA kappa	6 (14.3)	18 (13.7)	1.04 (0.38–2.88)	0.938
IgA lambda	4 (9.5)	16 (12.2)	0.75 (0.23–2.40)	0.623
Combined immunoglobulin isotype and light chain^e^				
IgG lambda	5 (15.6)	36 (37.1)	1.0 (reference)	
IgG kappa	27 (84.4)	61 (62.9)	3.23 (1.13–9.26)	0.029
Combined immunoglobulin isotype and light chain^e^				
IgA kappa	6 (18.2)	18 (22.8)	1.0 (reference)	
IgG kappa	27 (81.8)	61 (77.2)	1.35 (0.47–3.90)	0.580

^a^Laboratory characteristics were determined from serum

^b^Adjusted for sex, age, level of education (>high school graduate or equivalent) and race (White, Black)

^c^Cases with biclonal MM (*n* = 2), nonsecretory MM (*n* = 2), IgD MM (*n* = 1) were excluded from analysis. Cases with IgA MM served as the referent

^d^Cases with nonsecretory MM (*n* = 2) were excluded from analysis. Cases with lambda (l) MM served as the referent

^e^Cases evaluated included those with heavy-chain MM. Cases with light chain MM (*n* = 54) were excluded from analyses

## Discussion

MM is significantly more common in Blacks. However, our current understanding of MM is largely based on studies from patients of European origin. Thus, epidemiologic studies that include well-characterized MM patients from racially diverse populations are warranted to significantly improve our understanding of MM etiology and to provide a rationale for the differences observed in Black and White MM patients. To our knowledge, this is the first report of a comprehensive evaluation of the contribution of family history of hematologic malignancies and other cancers on the risk of MM, which included differences in Blacks and Whites and among cases, the presence of MM-defining clinical features. We observed a 3.75-fold increased overall risk of MM among participants who reported a family history of MM, and the effect was notably greater, by an order of magnitude, in Blacks than Whites (ORs 21 and 2, respectively), albeit our sample was small. In an evaluation of clinical features in MM cases with and without a family history of hematologic malignancy, anemia and renal insufficiency, attributed to MM, were less common, whereas hypercalcemia and lytic bone lesions were more common, albeit not significantly. In addition, we found a significant proportion of κ light chain restricted disease among MM patients with familial coaggregation of any hematologic malignancy.

The overall elevated risk of MM observed in our study is consistent with previous findings from case–control studies of patients with first-degree relatives with MM, yielding risk estimates ranging from twofold to sixfold [16, 17]. Our risk estimates are also similar to estimates generated from large, registry-based studies, where family history data were verified, thereby providing support for the validity and generalizability of our findings despite the possibility of bias in recalling cancer diagnoses in family members, which may differ by case–control status [25]. In the largest study published to date, which included 37,838 first-degree relatives of 13,896 patients with MM diagnosed in Sweden between 1958 and 2005, the risk of MM was increased 2.1-fold in first-degree relatives with MM (95% CI 1.6–2.9) [19], and in the Swedish registry study preceding this, the risk of MM was increased 4.25-fold (95% CI 1.81–8.41) [18]. In addition, Camp et al. [26] confirm this association in 177,226 first-, second-, and third-degree relatives linked to 1,354 MM patients included in the Utah Surveillance, Epidemiology and End Results (SEER) cancer registry. Findings originating from these large, population-based, registry studies yield precise estimates of association by virtue of providing sufficient statistical power; however, interpretations from registry-based studies thus far have been limited to persons of European origin.

Evidence for a stronger familial association of MM in Blacks observed in our study coincides with findings from the only population-based case–control study published to date, in which Brown et al. [20], report an elevated risk of MM in patients with affected first-degree relatives with MM of 17.4-fold (95% CI 2.4–348) in Blacks and 1.5-fold (95% CI 0.3–6.4) in Whites. Thus, despite the relatively small number of affected relatives with MM, strength and consistency of findings from this study and ours suggests a familial predisposition to MM, which is greater for Blacks than Whites. Together, these observations suggest that the excess familial risk of MM contributes, at least in part, to the overall increased incidence of MM observed in Blacks. However, because the frequency of familial MM in the general US population is low in both racial populations, germline susceptibility appears to contribute to only a proportion of the overall risk, emphasizing that both genetic and environmental factors play an etiologic role in this common complex disease.

Our observation that coaggregation of hematologic malignancies (i.e., NHL, HL, leukemia) in families of patients with MM occurs only in Whites, could suggest a common etiology of select lymphomas and leukemias in persons of European origin and conversely, specificity for a germline susceptibility to MM in Blacks. Several lines of evidence demonstrate a familial coaggregation of select hematologic malignancies and solid tumors in blood relatives of patients with MM [16, 17, 19, 26–28], suggesting a shared etiology. However, these studies have largely been restricted to populations of European origin. Positive evidence for a shared etiology with lymphoma and leukemia subtypes in Blacks has not been observed [20], perhaps due, in part, to the disproportionately lower incidence observed in this population.

In our analysis of solid tumors in blood relatives of patients with MM, we provide evidence for familial coaggregation of any solid tumor with MM in the combined population, consistent with prior reports [16, 19, 26]. In addition, we found modest non-significant evidence for a shared etiology with select tumor types previously shown to co-occur in families of MM (i.e., prostate, malignant melanoma, genitourinary cancers) [26], with the co-occurrence of malignant melanoma and genitourinary cancers observed only in Whites. Our observation of familial aggregation of head and neck cancers in relatives of MM patients among Blacks has not been previously reported. Additional studies are required to confirm this preliminary finding and to investigate a biological basis for a possible shared etiology.

Additional support for an inherited germline susceptibility arises from several gnostic and agnostic gene association and sequencing studies, which have been used to significantly expand the repertoire of confirmed MM susceptibility loci [29–31]. Despite recent advances in gene discovery, it is unknown how these MM loci influence the increased risk observed in Blacks because prior analyses have been conducted exclusively in populations of European origin. Further evidence for a germline susceptibility points to the Major Histocompatibility Complex (MHC) as a genomic region with sufficient allelic variation by race to account for the higher incidence of MM observed in Blacks [32]; however, findings from genome-wide association studies have not confirmed these relationships.

To our knowledge, this the first comprehensive case–control study used to evaluate the contribution of family history of any hematologic malignancy on the presence of defining clinical features and laboratory characteristics in MM patients. We hypothesized that MM patients with a stronger familial predisposition are more likely to present with clinical features and laboratory characteristics consistent with increased disease burden. Although we note a significantly younger age of MM onset and a modest non-significant increase in the presence of lytic bone lesions in MM patients with a familial coaggregation of hematologic malignancies, we did not observe differences in laboratory characteristics that are typically associated with disease burden including M-protein, abnormal FLC ratio, percent clonal bone marrow plasma cells, and β2-microglobulin, nor did we observe differences by the presence of cumulative organ damage or ISS staging. Lack of association with laboratory characteristics, typically associated with increased disease burden, may reflect inadequate statistical power to detect modest effects. However, we did observe a significant proportion of cases with κ light chain restriction.

One of the hallmarks of MM is the clonal proliferation of malignant plasma cells, which produce M-protein and cause lytic bone lesions. Because IgG is the most common isotype and κ is the most common light chain, which constitute the M-protein, we acknowledge the possibility that our finding could be due to chance. However, we did not observe an over-representation of the IgG isotype in MM patients with a familial coaggregation of hematologic malignancies, suggesting that light chain restriction may have a stronger familial component either by germline susceptibility or shared environment. Findings from a familial case series do not support a germline susceptibility to M-protein [33]. However, κ restriction in MM patients with a familial coaggregation of hematologic malignancies may reflect the impact of environmental exposures on a common genetic background capable of driving an antigenic-dependent process. In this capacity, antigen may play a role in selecting and expanding B cells, which eventually promote the monoclonal expansion of plasma cells with a predominant κ light chain restriction. Evidence for an antigenic-dependent process in the etiology of MM comes from findings that prior exposure to select pathogens and autoimmune disease are associated with MM risk [34–38]. Additional epidemiologic and molecular studies are warranted to confirm these findings and to elucidate the role of an antigen-dependent process in MM etiology.

This investigation was specifically designed to evaluate risk factors associated with MM and differences in well-characterized Black and White MM cases and matched controls. However, interpretation of our findings is not without limitation. Despite efforts to minimize the effect of recall bias by adjusting for factors related to the accuracy of self-reported family history and with the disparity in MM incidence by race (i.e., age, sex, race, education) [25, 39], residual bias resulting from potential differences in case–control reporting may lead to an overestimation of risk. However, the consistency of findings with previously published reports from population-based registry studies suggests that any potential bias was unremarkable. Although we do not anticipate differences in family size by case–control status or in Blacks and Whites in our region, we recognize the possibility that family size could influence the effect of family history of cancer on the risk of MM because larger families provide more persons at risk for disease. Finally, sample size and the inability to systematically obtain and validate family history data precluded our ability to evaluate familial coaggregation of leukemia subtypes by race and to delineate relationships of MM-defining clinical features, family history of MM in first-degree relatives and differences by race or other meaningful strata (e.g., early age of onset). Additional large, well-characterized and racially diverse populations, made available through multi-center cancer consortia, will be required to further delineate these relationships.

In summary, we confirm a positive association of familial risk of MM, which is greater in Blacks, and describe for the first time, variation in the presence of defining clinical features in MM patients according to family history of hematologic malignancy. Although we cannot exclude the possibility that our observed associations and patterns of inheritance might be due to chance, the consistency of our results supports a combined germline and environmental susceptibility. Our findings underscore the importance of further characterizing germline and somatic variation in addition to the mechanisms by which previous environmental exposures modify the genetic predisposition to disease [40–42] in similarly well-characterized racially diverse populations. Such characterizations may facilitate improvements in our ability to predict clinical progression, response to treatment and underlying biologic mechanisms.

## Electronic supplementary material

Supplementary material 1 (DOCX 21 kb)
