# Supplementary material for: Family history of hematologic malignancies and risk of multiple myeloma: differences by race and clinical features
Source: Cancer Causes Control. 2015 Nov 23;27:81–91. doi: 10.1007/s10552-015-0685-2 (PMC4703620; doi:10.1007/s10552-015-0685-2)
Supplement: Supplementary file 1 [file 10552_2015_685_MOESM1_ESM.docx]

Supplementary Table 1. Risk estimates of myeloma associated with an increasing number of affected relatives and sex of affected relative with any cancer, any hematologic malignancy and multiple myeloma, overall and stratified by race

	White				Black				Total Population	
	Case, N (%)	Control, N (%)	OR (95% CI)[a]	P	Case, N (%)	Control, N (%)	OR (95% CI)[a]	P	OR (95% CI)[b]	P
**Affected relatives with any cancer**										
Affected relatives[c]										
None	18 (11.7)	44 (16.7)	1.0 (reference)		21 (20.0)	62 (31.3)	1.0 (reference)		1.0 (reference)	
1	27 (17.5)	75 (28.5)	0.94 (0.45-1.98)	0.868	33 (31.4)	62 (31.3)	1.76 (0.90-3.44)	0.100	1.30 (0.79-2.14)	0.295
≥ 2	109 (70.8)	144 (54.8)	2.28 (1.20-4.32)	0.012	51 (48.6)	74 (37.4)	2.45 (1.30-4.65)	0.006	2.44 (1.56-3.81)	<0.0001
P trend				0.001				0.006		<0.0001
Male relative	103 (66.9)	148 (56.3)	1.90 (1.22-2.95)	0.004	59 (56.2)	78 (39.4)	2.37 (1.43-3.95)	0.001	2.03 (1.46-2.83)	<0.0001
Female relative	99 (64.3)	156 (59.3)	1.32 (0.86-2.02)	0.200	57 (54.3)	94 (47.5)	1.35 (0.84-2.19)	0.218	1.34 (0.97-1.84)	0.071
**Affected relatives with any hematologic malignancy**										
Affected relatives[c]										
None	113 (73.4)	217 (82.5)	1.0 (reference)		89 (84.8)	183 (92.4)	1.0 (reference)		1.0 (reference)	
1	35 (22.7)	38 (14.5)	1.82 (1.07-3.08)	0.027	13 (12.4)	13 (6.6)	2.25 (0.98-5.17)	0.055	1.87 (1.20-2.91)	0.006
≥ 2	6 (3.9)	8 (3.0)	1.59 (0.53-4.78)	0.411	3 (2.9)	2 (1.0)	3.63 (0.59-22.5)	0.166	2.03 (0.80-5.14)	0.868
P trend				0.038				0.023		0.004
Male relative	25 (16.2)	27 (10.3)	1.77 (0.98-3.22)	0.060	10 (9.5)	11 (5.6)	2.12 (0.86-5.26)	0.104	1.86 (1.14-3.06)	0.014
Female relative	12 (7.8)	13 (4.9)	1.74 (0.74-4.08)	0.206	8 (7.6)	5 (2.5)	3.11 (0.97-9.96)	0.056	2.02 (1.03-3.98)	0.042
**Affected relatives with multiple myeloma**										
Affected relatives[c]										
None	143 (92.9)	253 (96.2)	1. 0 (reference)		96 (91.4)	197 (99.5)	1.0 (reference)		1.0 (reference)	
1	11 (7.1)	9 (3.4)	2.27 (0.90-5.75)	0.082	6 (5.7)	0 (0.0)	-	-	3.75 (1.62-8.65)	0.002
≥ 2	0 (0.0)	1 (0.4)	-	-	3 (2.9)	1 (0.5)	7.50 (0.75-74.6)	0.086	3.84 (0.63-23.5)	0.146
P trend				-				-		0.001
Male relative	6 (5.7)	6 (2.3)	1.76 (0.54-5.69)	0.345	6 (5.7)	1 (0.5)	14.5 (1.71-123)	0.014	3.59 (1.38-9.34)	0.009
Female relative	2 (1.3)	2 (0.8)	2.19 (0.30-16.0)	0.442	5 (4.8)	1 (0.5)	10.7 (1.21-93.7)	0.033	5.15 (1.30-20.4)	0.020

[a]Adjusted for sex, age, and level of education (> high school graduate or less).

[b]Adjusted for sex, age, level of education (> high school graduate or less) and race (White, Black).

[c]Multiple cancers occurring in a single first-degree (parent, sibling, child), second-degree (grandparent, aunt, uncle, niece, nephew) or third-degree (first-cousin) relative were included as independent cancers. Therefore, total cancers may be greater than the total number of affected relatives.
